# Weight-Related Barriers for Overweight Students in an Elementary Physical Education Classroom: An Exploratory Case Study with One Physical Education Teacher

**DOI:** 10.3389/fpubh.2017.00305

**Published:** 2017-11-17

**Authors:** Mary Odum, Corliss W. Outley, E. Lisako J. McKyer, Christine A. Tisone, Sharon L. McWhinney

**Affiliations:** ^1^Department of Health and Human Performance, Texas State University, San Marcos, TX, United States; ^2^Department of Recreation, Park & Tourism Sciences, Texas A&M University, College Station, TX, United States; ^3^Department of Health Promotion and Community Health Sciences, Texas A&M Health Sciences Center, College Station, TX, United States; ^4^Department of Health and Kinesiology, Texas A&M University, College Station, TX, United States; ^5^Department of Agriculture, Nutrition & Human Ecology, Prairie View A&M University, Prairie View, TX, United States

**Keywords:** childhood obesity, elementary school, physical education, qualitative, thematic analysis, structural analysis

## Abstract

**Introduction:**

As physical performance may be more difficult for overweight children than for their non-overweight peers, understanding how weight impacts student performance in the physical education (P.E.) classroom could inform school-based obesity prevention programming.

**Materials and methods:**

This qualitative case study examined one elementary physical educator’s perspectives of overweight students’ weight-related experiences in her classroom. Narratives were elicited during an in-depth interview and analyzed using structural and thematic analyses. We utilized the social cognitive theory to inform our exploration of the narratives.

**Findings:**

The thematic analysis illuminated a behavioral pattern of student refusal to participate in the P.E. classroom while the structural analysis emphasized the teacher’s constructive, individualized responses to participation refusals. Combined, the two analytic techniques provided a more holistic snapshot of the experiences of overweight students in this elementary school. In addition, a preliminary model explaining the behavioral pattern among overweight students in this particular P.E. classroom was created.

**Discussion:**

Students who were overweight were more likely to initially refuse to attempt physical tasks in the classroom because they feared peer ridicule, and the teacher played a critical role in whether these students chose to participate in subsequent classes. As agents of change, P.E. educators should be included in formative stages of comprehensive, systemic changes to combat childhood obesity.

## Introduction

Physical education (P.E.) teachers are uniquely positioned to assist school-based obesity prevention given their training, expertise, and potential to influence student physical activity. While elementary school personnel, including P.E. teachers, have been routinely included in U.S. school-based obesity prevention efforts, their perceptions of, and recommendations for managing obesity has been largely absent from current research reports (1). The literature to date has often focused on the perceptions of school nurses (2–5), and less commonly include P.E. teachers (6, 7). As physical performance may be more difficult for overweight children than for their non-overweight peers, understanding how weight impacts student performance in the P.E. classroom could inform school-based obesity prevention programming.

The school environment has repeatedly been targeted for childhood obesity prevention because children spend a significant amount of time at school, schools are equipped with facilities and personnel to implement nutrition and physical activity programs, and schools afford access to students across various socioeconomic and demographic groups. However, the most recent School Health Policies and Practices Study results indicate the percentage of schools requiring students to take P.E. as a requirement for graduation or advancement to the next grade level has decreased from 96.4 to 76.5% between 2000 and 2014 (8). During this same time frame, incidence of obesity among school-age children in the U.S. increased.

While some epidemiological reports indicate overall childhood obesity rates are plateauing in the U.S., the most recent National Health and Nutrition Examination Survey has indicated an obesity rate of 17.5% among U.S. school children aged 6–11 years, with varying prevalence by race/ethnicity: non-Hispanic Asian (9.8%), non-Hispanic white (13.6%), non-Hispanic black (21.4%), and Hispanic (25.0%) (9). Extreme obesity, defined as at or above the 120% of the sex-specific 95th percentile on the CDC BMI-for-age-growth charts, was 5.6% with a similar racial/ethnic pattern: non-Hispanic Asian (1.0%), non-Hispanic white (3.4%), non-Hispanic black (8.8%), and Hispanic (9.3%) (9).

The negative health consequences of childhood obesity have been well documented in a large body of prior work. Previous studies have established health complications associated with obesity during childhood, including significantly higher all-cause mortality (10, 11), heart disease morbidity and mortality (12–14), metabolic syndrome (11, 14), and overall decreases in quality of life, self-esteem, and physical functioning (15). In the U.S., the importance of addressing childhood obesity with physical activity is underscored with Healthy People 2020 objectives (16, 17) and Centers for Disease Control and Prevention recommendations (18).

For school-based programming to effectively combat childhood obesity and its comorbidities through P.E. programming, P.E. teachers’ expertise and insights should be utilized throughout all stages of program planning, implementation, and evaluation. To this end, this study presents formative data collected during a program needs assessment informing the development of a school-based obesity prevention program. Specifically, this study presents an elementary P.E. teacher’s perspectives on the impact of weight on overweight students’ experiences in her classroom.

The purpose of this study was twofold. First, we investigated if overweight elementary students faced weight-related challenges in one elementary P.E. classroom. To this end, we elicited narratives from a veteran P.E. teacher about her students’ weight-related experiences. Second, we utilized multiple data analytic methods on these narratives to determine whether a combination of analyses revealed unique findings compared to a single analytic technique. Our overarching research question was, *what are the weight-related experiences of overweight children in the P.E. classroom as described by an experienced P.E. teacher?*

## Materials and Methods

### Narrative Framework

We selected a narrative framework for multiple reasons: 1. storytelling is a natural communication method; 2. narrative analytic methods are varied and provide insight into both *what* is said and *how* it is said; and 3. narratives are useful for identifying problems.

Storytelling is a natural communication method for humans; it is how we “make sense of our experiences, how we communicate with others, and through which we understand the world around us” [(19), p. 32]. In addition, stories (narratives) pass along traditions from generation to generation; they are the “coin and currency of culture” [(20), p. 16]. We invited our participant to share stories about the experiences of overweight elementary students in her P.E. classes so that we could access the “currency of culture” related to the impact of weight on overweight elementary students in the P.E. setting. Thus, our participant was able to share her experiences—namely, her perception of the experiences of her overweight students—*via* the familiar communication method of storytelling.

Narratives also provide researchers with data that can be studied in larger “chunks” than in some other qualitative methods. Analyzing intact stories affords researchers the opportunity to study not only the *content* of the data but also the *form* of the data, the structure in which the stories are told. Merriam (19) explains that narrative analyses have an “emphasis on the stories people tell and on how these stories are communicated—on the language used to tell the stories” (p. 202). This study utilized two analytic techniques: 1. a thematic analysis to investigate the content; and 2. a structural analysis to evaluate the form and structure of the narratives. These multiple lenses through which the data were filtered strengthen the richness of data interpretation.

In addition, narratives are useful for identifying problems, as Bruner (20) observed: “Great narrative is an invitation to problem finding, not a lesson in problem solving. It is deeply about plight, about the road rather than about the inn to which it leads” (p. 20). Our intent was to identify potential weight-related problems faced by overweight elementary students in one P.E. classroom to inform the empirical literature and future research and intervention endeavors.

### Participant and Setting

At the time of this study, the participant was the sole P.E. teacher in a rural elementary school in a southwestern U.S. location. The participant volunteered for this study because her school was part of a multi-year childhood obesity prevention project, and she was interested in providing insight during the needs assessment to inform intervention development. At the time of data collection, the participant had 15 years of teaching experience: Seven years as a high school teacher and coach and eight as an elementary P.E. teacher. She was Caucasian, female, in her early 40s, and had earned a Bachelor’s degree.

At the time of data collection, the elementary school served approximately 500 students from kindergarten to fifth grade, all of whom had 135 min weekly in the participating teacher’s P.E. classroom. P.E. facilities on the school campus included a large indoor gym, an outdoor track, and open land space for team sports like soccer. In accordance with school district policy, the teacher used the Coordinated Approach to Child Health curriculum (21).

The racial/ethnic profile of students at the school was 64.4% Hispanic, 24.2% Black, and 11.4% Caucasian. According to school district data, in the school year preceding this study, 26% of third-grade students were overweight according to the BMI measures taken as part the school’s wellness plan, which was approximately 1.5 times higher than the corresponding nationwide percentage of 17% for the same year. School personnel participating in other portions of this needs assessment confirmed obesity was prevalent among their students, and expressed a desire to be part of a school-based obesity prevention program (7).

### Instrument

Utilizing an interview guide approach (22), the participant was asked open-ended questions about weight-related barriers students faced in the P.E. classroom (e.g., How does a child’s weight affect her/his ability to participate in your P.E. class? How do classmates react to overweight children both inside and out of the P.E. classroom?). As we did not have access to individual student’s BMI measures (just the percentages for the third-grade students measured in the preceding year by the school district and referenced previously), the identification of overweightness among students discussed in this study was up to the teacher’s perception. Beyond the interview protocol, *ad hoc* questions were asked in response to ideas that arose during the interview and the participant was encouraged to add additional thoughts and follow-up on previous responses.

### Procedure

At the participant’s request, we conducted the interview on her school campus. The interview was audio-recorded and transcribed into a verbatim transcript with cleaned speech (23). Field notes were taken by the interviewer to supplement transcription and data analysis processes. To protect the identity of our participant, we used a pseudonym. To protect the identities of students in her classroom, we asked the participant to use pseudonyms when telling her stories. The participant signed a written informed consent form and was compensated with a $50 gift card to a local retailer. All study procedures were approved by the Texas A&M University Institutional Review Board.

### Data Analysis

We reviewed the raw interview data to establish the best way to divide our data into distinct narratives. We divided the data into 14 narratives, one for each student the participant (who we’ll call “Laotha”) discussed. Six narratives focused on female students and eight focused on male students. Typically, each story was shared intact (complete from start to finish), but on occasion the participant referred back to a story to provide more insight on a particular student. We handled these cases by adding the supplemental data shared later in the interview to the already established narrative of that particular child, resulting in one complete narrative for each student.

With 14 distinct narratives defined, we conducted constant comparison (24) and structural analyses (25, 26). In a team of three, we used the constant comparison method to separate and categorize recurrent or significant themes across the 14 stories until 100% consensus was reached on codes. We also utilized social cognitive theory to inform our thematic exploration of narratives: specifically, we used the model of reciprocal determinism to identify personal, environmental, and behavioral determinants impacting student behavior (27).

We employed structural analysis to determine how stories were told, to identify the participant’s storytelling structure, and to analyze intact narratives, which helped illuminate findings not observed with the thematic analysis. Namely, it enabled us to identify the structural breakdown of the narratives; to organize the narratives by Labov and Waletzky’s (28) six elements (abstract, orientation, complicating action, evaluation, resolution, and coda). While the structural analysis illuminated some new ideas, it also reinforced themes from the thematic analysis, highlighting the usefulness of using these two complementary analyses together for our data.

## Findings

### Theoretical Framework

We utilized Albert Bandura’s (27) social cognitive theory to inform our exploration of Laotha’s narratives, which espouses a model of causation in which personal, environmental, and behavioral determinants reciprocally interact, each affecting the other. Laotha’s stories about the experiences of overweight children in her P.E. classroom contain elements of all three determinants in an interactive cycle. For example, consider the narrative about “Riley” (all student names are pseudonyms), one of Laotha’s overweight students:

Participant: I had one student [who] totally refused to do the unit [P.E. lesson], at all, during class. And so, what I had to do was, I made an arrangement with [his] parent for him to come after school and do the unit just with me. And just to try with me. And once I got him into the skills and rolling, and doing some of the jumps, then he was okay to join the class the next week. But that first week, he refused to do it, just because he was embarrassed and it was difficult for him. But when he worked with me after school and saw that, really, hey, I really can do this, then we were okay…
Interviewer: Did he give you any reasons why he wouldn’t want to try the activity in class, why he was embarrassed?
Participant: Because, ‘I don’t want everybody looking at me’ and ‘I can’t.’ ‘I can’t’ was the biggest, and ‘I don’t want everybody looking at me’; those were the two biggest…I told [Riley]; ‘listen, Honey, nobody’s looking at you, they’re worried about doing their own, you know? They’re not worried about you…He was just afraid that his buddies, ’cause he was one of the tough guys, so, ‘clearly, I’m not going to embarrass myself in front of my buddies,’ you know? And, so, he was convinced that that was going to be the [reaction] and he was going to lose some status. Keeping him after school was the good thing to do there just because we got him into a position where he knew he wasn’t going to be embarrassed or defaced in front of his friends.

This example highlights the reciprocal determinism among Riley’s beliefs, behavior, and environment. As Laotha shared, the “biggest” reason Riley initially refused to attempt the assigned P.E. task in the classroom was his lack of confidence in his ability to successfully complete it, that is, his lack of *self-efficacy* to complete the given P.E. task (29). This lack of self-efficacy (personal determinant) contributed to his decision not to participate in class (behavioral determinant), partially because it triggered a fear of peer judgment and loss of status (personal determinants): “clearly, I’m not going to embarrass myself in front of my buddies.” The daunting P.E. classroom with peer spectators (environmental determinant) provided further incentive for Riley’s participation refusal. These three reciprocally interacting determinants all contributed to Riley’s refusal to participate.

Laotha facilitated Riley’s participation by wisely changing one of the determinants, the environment. By working with Riley in a setting without classmates, Laotha successfully removed his second “biggest” rationale for refusal—“I don’t want everybody looking at me”—and worked with him in the more comfortable environment *until his self-efficacy was increased*, and he was in a “position where he knew he wasn’t going to be embarrassed or defaced in front of his friends.” In this new environment, Riley was willing to attempt previously formidable P.E. tasks, which increased his self-efficacy for the tasks and gave him the confidence to attempt the activity in the classroom setting alongside his peers.

Reciprocal determinism demonstrates the power of the interaction of behavioral, personal, and environmental determinants on human behavior. Each of Laotha’s 14 narratives provided clear evidence of the power of reciprocal determinism on the behavior of her overweight students, which is why we used the framework for our analyses.

### Thematic Analysis

Three major themes emerged: 1. lack of self-efficacy to perform P.E. tasks; 2. fear of peer rejection; and 3. constructive manipulation of determinants to change behavior.

#### Lack of Self-Efficacy to Perform P.E. Tasks

A lack of self-efficacy was evident in all 14 narratives. Our participant reported the most commonly used excuse by overweight students to evade P.E. activities was some variation of “I can’t.” She described numerous situations in which overweight students initially refused to attempt a P.E. task because they lacked confidence in their ability to successfully complete it. In particular, excuses were made more often for activities that involved gymnastics or running (e.g., basketball, soccer, and track). Some excuses given by students included, “I can’t do it, because I’ve never done that before. I just can’t”; “I can’t run! I can’t run!”; “I’m no good at this game”; “I can’t do it because I’m bigger, I can’t run as fast and when I do run, I’m gonna fall and slip.” Although non-overweight students avoided P.E. tasks on occasion, overweight students were more likely to avoid P.E. activities and were also more likely to “struggle with that attitude over and over, ‘I can’t’ and ‘I won’t’ and ‘no.’”

#### Fear of Peer Rejection

The fear of peer judgment was present in all 14 narratives. Overweight students were more likely to cite fear of peer ridicule (personal determinant) than their non-overweight counterparts. Some variation of “I don’t want everybody looking at me” was regularly used by overweight students to evade a new or difficult P.E. task, and was the second most common evasion excuse reported.

Overweight students were afraid of losing social acceptance if they unsuccessfully attempted a P.E. task in front of peers. The participant believes this fear is misdirected: she identified that the issue begins when peers see a classmate not participating in required activities. The underlying motive of a peer “telling on” those not participating (regardless of weight) was fairness: Every classmate *should* be participating and when one does not s/he attracts the attention of and is “called out” by peers. Because overweight children were more likely to refuse to participate, they were singled-out more often than their non-overweight counterparts:

Participant: The kids really don’t care about their weight at all if they’re helping out, if they’re participating. It really is amazing; kids are kinda a little blind in that area because they’re all so many different sizes. I mean, in the same grade, I have a 5th grader that’s really small for his age and then I have one who looks like he’s going into 8th grade. So, they’re so many different sizes. And so those little guys are really good about not discriminating, be it weight or color or anything else, really. They’ll talk about each other’s mamas but they don’t discriminate. As long as they’re playing, and having fun, then we really don’t struggle with that kind of thing. But the struggle comes when they’re not doing what they’re supposed to do. Or, if we’re doing stations, I’ll have little people come tell me, ‘Hey, Sophie’s not doing so-and-so’ or ‘Sophie won’t …’ you know? So, they’ll tell on the bigger kids if they’re not participating.
Interviewer: Would they do that if another ‘little person’ wasn’t participating?
Participant: Oh yeah, Anybody. I know everything [laughing] because they’re gonna tell. They’re gonna tell to me. If they think I don’t see it, they’re gonna let me know. [laughing]. Yeah, it’s not just the big kids that get told on. But, it’s more prevalent just because they’re usually the ones that are not putting forth a lot of the effort. Just because, I think they are scared that they’re not going to be able to do it as well.

#### Constructive Manipulation of Determinants

The third major theme was rooted in the participant’s response to student participation refusals. First, she would determine the underlying cause for the avoidance (often low self-efficacy or fear of peers watching). Second, she would engage with the student in an attempt to build up their confidence (personal determinant) to attempt the task in the class. If refusal continued, she would reduce the barriers. For example, in several narratives, she removed the peer intimidation factor by meeting with the student outside of class time (environmental determinant) to work one-on-one on the physical task until that student was efficacious enough to attempt the task in class. Another common approach was breaking down the task into more manageable goals, to build student confidence.

Consider the story of “Rebekah,” a fourth-grade girl who dreaded running so much she successfully evaded her required 1-mile run test for 2 weeks: The first week with a complaint of knee pain and the second week by wearing her house shoes to school. During the 2 weeks of non-participation, the participant encouraged Rebekah, in an attempt to increase her self-efficacy (personal determinant). In the third week, the participant set a more manageable goal of jogging the first lap and then walking the rest of the mile (environmental determinant):

Participant: She ran—she jogged the first lap until she got to me, which was about a fourth of a mile, which is significant for her, and she did jog it, the first one [lap]. After that, she only jogged the last part when I was yelling, “Come on! You’re almost done! Run!” And then she ran the last 50 yards.
Interviewer: How did she feel when she finished?
Participant: Much better. Now, after she finished, that was a great teaching moment because I said, “[Rebekah], look, you got it in–” I think she ran it in like 18:39 or something like that. It was not a great time, but for her it was a huge accomplishment, so yeah, we celebrated. I was like, “Look at what you did!” and “This is great! You broke your record today! Look on your card!” We have running cards that we punch every time you run, and usually she goes two or three laps only. This day because of her mile run by the end she had done her four, which was almost the mile, and then that fifth one, which completed the mile, so she had five! And so, I said, “You set a new record today!” And so, she was smiles and I said, “[Rebekah], do you see how that feels? I want you to remember how this feels today because this is the way it feels every day in P.E.! If you’ll do your best, if you’ll really try, this is the way it feels.” You know? She said, “Ok.” You know she’s good, but it did not transfer immediately over. But the good news is that I can use that time to point back and say, “Ok, remember [Rebekah]? Remember right then? Remember how you felt? Let’s try that again today.”

### Structural Analysis

We categorized the bulk of the narratives as *complicating action*, but noticed two distinct foci within this element: (1) the struggles of students who are overweight that led to their non-participation and (2) the teacher’s subsequent intervening to encourage participation (most commonly through manipulating an environmental determinant). The distinctiveness of these subsections led us to separate *complicating action* into two categories: *complicating action* to denote student action and *intervention* to denote teacher action.

The *intervention* always came after *complicating action* and typically led to a *resolution* and/or *coda*. *Intervention* identifies the teacher’s customized approach to student participation refusals to encourage subsequent participation. In the narrative about “Zelda” (Table 1), the *intervention* led to a constructive *resolution*: “She did finally attempt” (line bb). In the narrative about “Nathan” (Table 2), the *resolution* was less positive: “I put Nathan in time out for a minute and talked to him again” (line kk).

**Table 1:** Structural analysis of narrative about “Zelda.”

**Abstract** a. (Interviewer) *Can you think of a particular child, and an example with gymnastics?* **Orientation** b. Sure, let’s call her “Zelda!” [laughing]. c. Zelda was struggling with the forward role d. and she was a fourth grader. e. Not obese, but overweight. **Complicating Action** f. And she would not try it; she just refused to try. g. So, she would stand against my whiteboard while the other classmates are going. h. She did this during that whole class period i. after I even talked to her repeatedly, just asked her to try whatever, j. and there was no going. k. So, after class—when everybody else was getting their drink—I just pulled her to the side l. and I said, ‘okay, let’s fix it. What can we do?’ m. And what I found out n. from talking to Zelda o. was that one thing was in her group there were no other people as big as she was, or even close. p. The ones in her group were mostly some smaller ones, q. there were a couple of ones that were regular sized r. but she didn’t want them to be looking at her. Intervention* (author’s addition to Labov and Waletzky categories) s. So, what I did was, for the next class, I just switched her and put her in another row t. before she came in, so it wouldn’t even be noticeable to this student [Zelda], u. I told her which row to go in and v. she sat in a different row with a different team and w. in that row there was a young man and another young lady x. that sat one in front and one behind her **Evaluation** y. that were not quite as big a she was z. but they were some of my bigger kids, too. **Resolution** aa. So, when she saw them trying and going, bb. she did finally attempt. cc. It still took us, what [counting] one, two, three, four stations dd. for her to try it, ee. but she was more willing ff. when she was surrounded by others that were not just tiny and flipping and, you know, going crazy.

**Table 2:** Structural analysis of narrative about “Nathan.”

**Abstract** a. (Interviewer) *How do ‘normal-weight’ kids react to overweight kids in your classes?* **Orientation** b. today we had a little soccer time c. and Nathan was not playing with his team, d. he was just standing at one end of the field by the goalies. Intervention* (author’s addition to Labov and Waletzky categories) e. So, I walked over there f. and said, ‘You know, Nathan, buddy, you’re not helping your team. g. Your team needs ya, you know?’ (…) h. And I said, ‘Alright, Nathan, buddy, we’ve got to get in there, i. you’ve got to help your team. j. Because, your team’s looking back and you’re back here and they need ya!’ (…) **Complicating Action** k. But he said, ‘I ain’t playing in this game. l. I’m not good at this game. m. I don’t wanna play this game.’ Intervention* (author’s addition to Labov and Waletzky categories) n. As so I went with the whole, o. “Honey, in Math class, we have to do math whether we like math or not. p. We have to do it. q. And then we learn that we like math cause it’s fun.” r. I said, ‘you gotta play the soccer game to figure out you like it. s. It really is fun! You know? t. Get up there with you team.’ **Complicating Action** u. And the other guys were playing, playing, playing, v. didn’t even see me chatting with him. w. But I left and went back over just kinda in the middle and watching everything x. and I saw one of my little guys go back and say, ‘Come on in! We need you!’ y. You know, whatever he’s saying to him (…) z. And then the little guy, let’s call him “Zeek”, aa. Zeek turns around and looked at me and goes [throws hands in the air]. bb. (Interviewer) *Like, what’s up??* cc. Yeah. [as if to say] ‘You know, he’s not even helping me out! What’s going on?’ dd. And, so, I just said, ‘Go play, go play.’ ee. And, so I just left him [Nathan] there for another few minutes (…) ff. he did scoot up, gg. and he did kick a ball that would come to him hh. but he did not approach, ii. he did not assert himself. (…) **Evaluation** jj. So, it’s not happening (…) it did not improve from that point. **Resolution** kk. I put Nathan in time out for a minute and talked to him again. **Coda** ll. We’ll have to deal with that problem on another day, mm. because we did not solve that one today.

By focusing on *how* our participant structured her stories rather than just *what* she recounted, we discovered that (1) her stories were intently focused on her students, (2) she downplayed her role in student participation successes, and (3) her interventions were vital for, but did not predict, student participation. The *intervention* sections focused on the participant’s action and were much shorter that the *complicating action* sections highlighting student action.

Our participant consistently kept the focus of her narratives on her students; their fears, their interaction with other students and with her, and their responses to her interventions. For example, she portrayed Zelda as the lead character while and downplayed her vital role of environmental manipulation that ultimately led to Zelda’s attempt of the P.E. activity. Consistently, our participant downplayed her role in student participation: She gave students credit for their decisions to participate, framing it as a student success. However, she readily accepted responsibility when students refused to participate and took action to engage them.

Structural analysis findings were congruent with thematic discoveries and highlighted the teacher’s response to non-participation: She altered the situation by manipulating one of the three behavioral determinants—most often an environmental one. This approach was almost always successful in coaxing students to rejoin the class, making her presence critical to the engagement of overweight students in this classroom.

## Discussion

Our parallel use of thematic and structural analyses provided richer, more in-depth insight than the application of either method alone. Our thematic analysis illuminated a behavioral pattern of overweight students’ participation refusals while the structural analysis emphasized the teacher’s responsive intervention focused on individual students. Together, these analyses provided a more holistic snapshot of overweight students’ experiences in this P.E. classroom.

**Figure 1:**
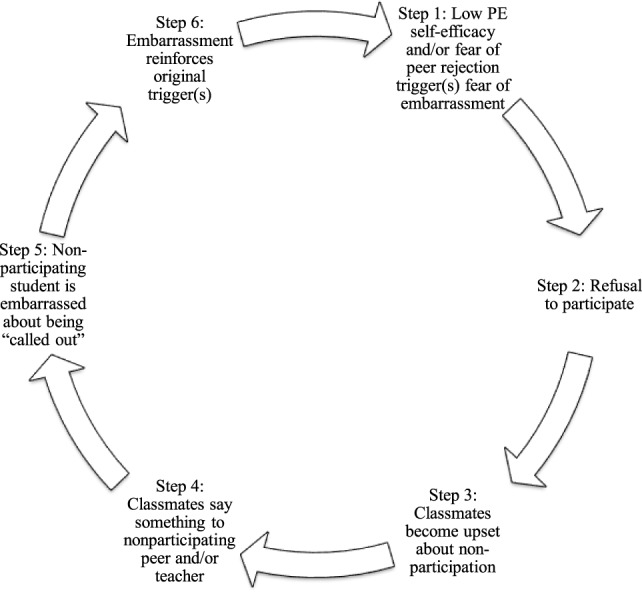
Behavioral pattern among overweight students in the physical education classroom.

During the thematic analysis, a pattern of behavior emerged associated with student participation refusals (see Figure 1). The pattern began when an overweight student was afraid of being embarrassed and/or had a low self-efficacy for a given physical activity. Consequently, a refusal to participate ensued. When peers noticed the non-participation, they became upset, said something to the non-participating peer and/or said something to the teacher. Being called out by peers was embarrassing for the non-participating student, which reinforced the original fear of embarrassment, thus continuing the cycle.

Interestingly, the participant reported overweight students’ lack of self-efficacy only for performing new P.E. tasks; not for previously attempted ones. We anticipated resistance to previously attempted P.E. activities that resulted in failure (i.e., inability to complete the activity), as self-efficacy has been found to mediate physical activity (30), or because they had experienced bullying, weight bias, or social stigma in the classroom, as found in prior research (31, 32). However, unlike previous findings that child obesity, itself, was a root cause of peer rejection (2, 3, 6, 33, 34), we found that non-participation in P.E. activities caused peers to focus on their overweight classmates, not their weight.

The structural analysis highlighted the teacher’s customized *intervention* for each non-participating student in an attempt to break the cycle of refusal. Evaluation of all 14 narratives revealed the teacher’s interventions were purposeful, constructive, and tailored for each student. These interventions typically occurred after classmates said something to their non-participating peer or to the teacher about their peer’s non-participation. During her intervention, the teacher manipulated at least one of the reciprocal determinants (personal, behavioral, and environmental) to encourage participation.

The teacher’s use of constructive manipulation of determinants allowed students to be active, intentional contributors to their actions and environments; therefore, *the decision that led to breaking the cycle was ultimately up to the student*. In other words, she intentionally supported student realization of human agency by allowing them to *play a part in their own self-development*. This approach is reflective human agency, which Albert Bandura (35) describes as allowing people to “play a part in their self-development, adaptation, and self-renewal with changing times” (p. 2). This agentic perspective of human behavior posits that people can intentionally exert control over their own actions (36), rather than being mere products of the environment. If the student’s refusal continued, s/he remained in the cycle. However, if s/he decided to participate, the embarrassing situation was diffused; her/his self-efficacy improved, and s/he was more likely to participate in the future.

While it is apparent the teacher’s interventions were vital for student participation, students did not always respond to the initial intervention and a select few students did not respond to multiple interventions. How many attempts it took a student to break the cycle varied, if it was ever broken. Some children broke it on the first try (e.g., Zelda); some needed multiple interventions (e.g., Rebekah); and others still had not broken the cycle, yet (e.g., Nathan). We failed to detect any difference in the teacher’s interventions to explain the direction and/or speed of students’ cycle breaking decisions. Future studies might investigate psychosocial variable impacting overweight elementary students’ decisions to participant (or not participate) in physical activity tasks in the P.E. classroom. Understanding—from the student perspective—reasons for avoidance of physical activity tasks would inform interventions to increase participation among students who are overweight.

Physical education teachers are trained and qualified to be an integral part of child obesity interventions and are uniquely positioned as “agents of change” to encourage the development of physical activity habits (37, 38). However, despite the position of the Institute of Medicine (39) that P.E. be a core component of public education, P.E. curricula have been historically at risk of exclusion from school programs and teachers have been excluded from the development of systemic reform efforts (40). We recommend P.E. teachers be included in the formative stages of school-based obesity prevention programs alongside school nurses. The student–teacher relationship established in the P.E. classroom may lead to conversations about weight, overcoming the perceived difficulty of establishing relationships with students reported by school nurses (5).

This study found that the P.E. teacher positively impacted her overweight students’ performance in her P.E. classroom. Specifically, we found the teacher–student interaction was the determining factor of student success in most of the narratives presented. Lessons learned from the teacher in this study may be useful for other elementary P.E. teachers: 1. reinforcing the autonomy of students, 2. encouraging and cheering for student participation/success, and 3. making accommodations (e.g., providing an alternative setting for initial attempts; providing smaller, incremental goals to achieve while working toward the larger goal) to foster an environment in which overweight students feel more comfortable engaging in physical activities. These recommendations align with recently published recommendations (41, 42).

### Limitations

Limitations include the inherent qualitative methodology constraints, including time and context. While our participant provided rich, robust narratives about students, the data only include the teacher’s perspectives, not those of the students in focus. In addition, given the voluntary nature of research participation, results may be biased by the perspective of a teacher who is willing to share teaching approaches and experiences as compared to teachers who would decline to participate. Despite these limitations, this study provides insight into experiences of overweight students in the elementary P.E. classroom which may inform future childhood obesity research and practice.

### Conclusion

This qualitative study explored the experiences of students who are overweight—from the teacher’s perspective—in one elementary P.E. classroom. We found that students who are overweight were more likely to initially refuse to perform physical tasks in the classroom due to fear of peer ridicule which, ironically, garnered negative peer attention. We also found that personal, behavioral, and environmental determinants impacted student behavior, as explained by social cognitive theory. Finally, we found that the teacher played a critical role in whether these students continued avoiding the behavior or chose to participate, underscoring the need of P.E. teacher inclusion in obesity prevention efforts.

## Ethics Statement

All study procedures were approved by the Texas A&M University Institutional Review Board with written informed consent from the participant.

## Author Contributions

MO—study design, data collection and analysis, manuscript preparation. CO, EM, CT, and SM—study design, manuscript preparation.

## Conflict of Interest Statement

The authors declare that the research was conducted in the absence of any commercial or financial relationships that could be construed as a potential conflict of interest.
